# Mycoviruses in *Aspergilli*: A Comprehensive Review

**DOI:** 10.3389/fmicb.2017.01699

**Published:** 2017-09-06

**Authors:** Ioly Kotta-Loizou, Robert H. A. Coutts

**Affiliations:** ^1^Department of Life Sciences, Imperial College London London, United Kingdom; ^2^Department of Biological and Environmental Sciences, University of Hertfordshire Hatfield, United Kingdom

**Keywords:** mycovirus, *Aspergillus*, population study, mycovirus classification, mycovirus transmission, hypervirulence, hypovirulence

## Abstract

Fungi, similar to all species, are susceptible to viral infection. *Aspergillus* is arguably the most well studied fungal genus because of its medical, ecological and economical significance. Mycoviruses were initially detected in *Aspergillus* species almost 50 years ago and the field continues to be active today with ground-breaking discoveries. The aim of the present review is to cover the scientific progress in all aspects of mycovirology as exemplified by *Aspergillus*-focused research. Initially an overview of the population studies illustrating the presence of mycoviruses in numerous important *Aspergillus* species, such as *A. niger, A. flavus*, and *A. fumigatus* with be presented. Moreover the intricacies of mycovirus transmission, both inter- and intra-species, will be discussed together with the methodologies used to investigate viral dispersion in a laboratory setting. Subsequently, the genomic features of all molecularly characterized mycoviruses to date will be analyzed in depth. These include members of established viral families, such as *Partitiviridae, Chrysoviridae* and *Totiviridae*, but also more recent, novel discoveries that led to the proposal of new viral families, such as Polymycoviridae, Alternaviridae and, in the context of the present review, Exartaviridae. Finally, the major issue of phenotypic effects of mycoviral infection on the host is addressed, including aflatoxin production in *A. flavus*, together with growth and virulence in *A. fumigatus*. Although the molecular mechanisms behind these phenomena are yet to be elucidated, recent studies suggest that by implication, RNA silencing may be involved.

## Introduction

**Figure 1:**
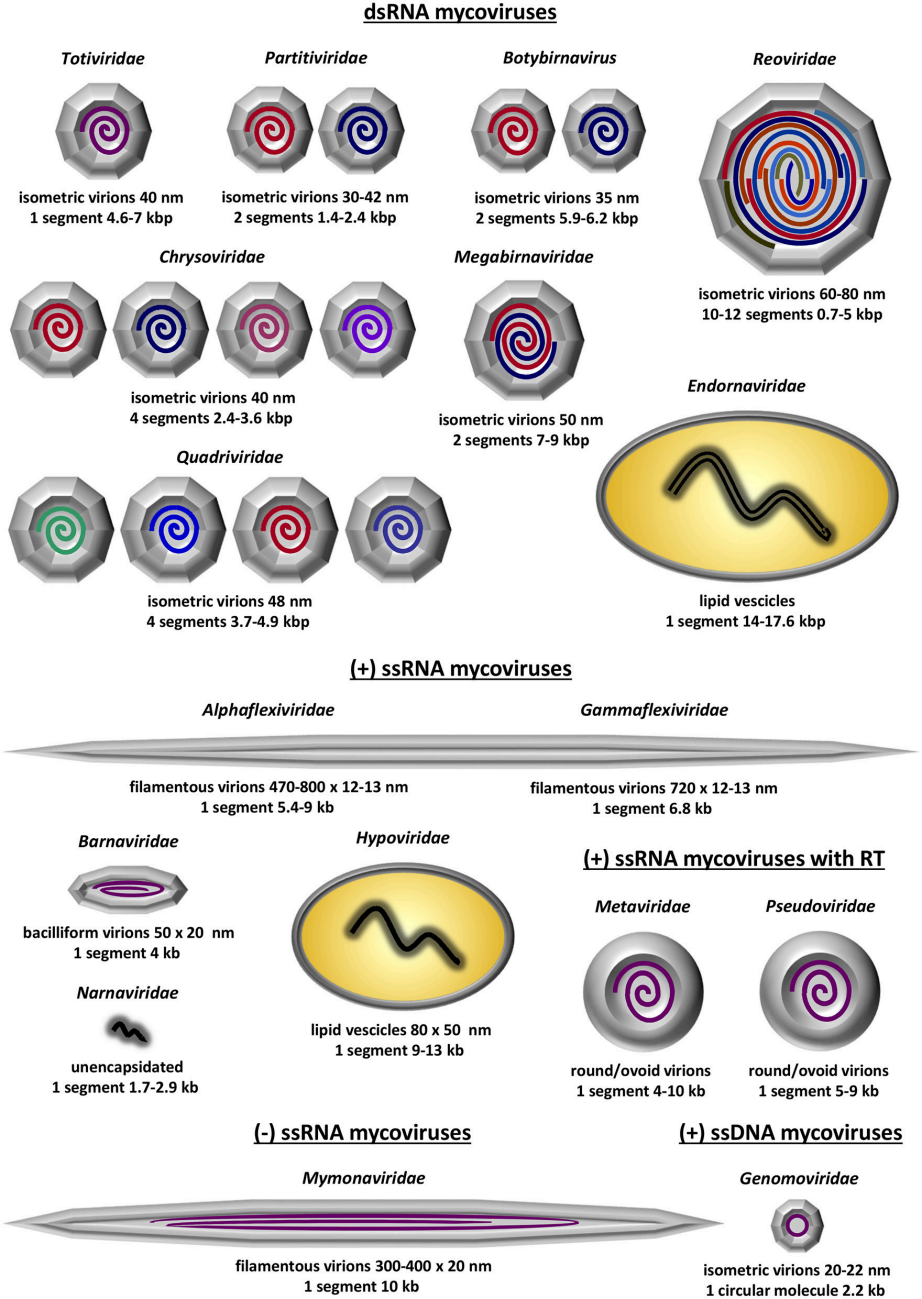
Schematic representation of the 13 established mycovirus families; 6 families containing dsRNA as genetic material, 5 families containing ssRNA as genetic material and 2 families containing ssRNA that is reverse transcribed. Depicted is the virion structure and number of genomic segments for each family. The information was derived from Virus Taxonomy: The Classification and Nomenclature of Viruses—The Online (10th) Report of the ICTV; https://talk.ictvonline.org/ictv-reports/ictv_online_report/.

The diversity of known fungal viruses, or mycoviruses, has increased rapidly over the last few years and this trend is expected to continue, especially with the development and the widespread use of state-of-the-art RNA deep sequencing techniques. Currently, the International Committee for the Taxonomy of Viruses (ICTV) officially recognizes 17 taxa, 16 families and one genus that does not belong to a family; these accommodate (exclusively or not) mycoviruses with double-stranded (ds) RNA linear genomes, positive-sense, single-stranded (ss) RNA linear genomes including reverse transcribing RNA linear genomes, negative-sense ssRNA linear genomes or ssDNA circular genomes (Figure 1). No mycoviruses with dsDNA genomes have been fully characterized at the molecular level yet. Since the majority of mycoviruses do not have an extracellular phase in their replication cycle, often their genomes are unencapsidated or non-conventionally encapsidated and true virions, if present, are proteinaceous in nature. To date, enveloped virions have not been discovered.

The dsRNA mycoviruses are the most commonly found and are classified in seven families and one genus: (1) *Totiviridae* (*totus* = “whole” in Latin) have non-segmented genomes and icosahedral virions. Genera *Totivirus* and *Victorivirus* infect exclusively fungi, while other totiviruses infect protozoa. (2) *Partitiviridae* (*partitus* = “divided” in Latin) have two genomic segments, each one encapsidated separately in distinct icosahedral virions. Genus *Gammapartitivirus* infect exclusively fungi, genera *Alphapartitivirus* and *Betapartitivirus*, infect both plants and fungi, genus *Deltapartitivirus* exclusively infect plants and genus *Cryspovirus* infect protozoa. (3) *Megabirnaviridae* (*megas* = “large” in Greek + *bi* = “two” in Greek + RNA) have two genomic segments, encapsidated together in icosahedral virions. The sole genus in the family exclusively infects fungi. (4) *Botybirnavirus* (*boty* from *Bot**r**y**tis*, the host fungus, + *bi* = “two” in Greek + RNA) have two genomic segments encapsidated in spherical virions and exclusively infect fungi. (5) *Chrysoviridae* were first isolated from the ascomycete *Penicillium* *chryso*g*enum* and have four genomic segments. Similarly to partitiviruses, each one of the four segments is encapsidated separately in distinct icosahedral virions. Chrysoviruses infect exclusively fungi. (6) *Quadriviridae* (*quattuor* = “four” in Latin) have four genomic segments encapsidated in spherical virions and the sole genus exclusively infect fungi. (7) *Reoviridae* (*r*espiratory *e*nteric *o*rphan viruses) have 10–12 segments, which, unlike partitiviruses and chrysoviruses, are encapsidated together in a large icosahedral virion. Out of the fifteen genera comprising the family, only genus *Mycoreovirus* infect fungi while the rest infect plants, algae, invertebrates and vertebrates including humans. (8) *Endornaviridae* (*endos* = “within” in Greek + RNA) possess non-segmented genomes that are non-conventionally encapsidated and accommodated in host-derived lipid vesicles. Both genera *Alphaendoviridae* and *Betaendoviridae* infect fungi, while the former also infect plants and oomycetes.

The positive-sense ssRNA mycoviruses all accommodate non-segmented genomes and are classified into five families: (1) *Alphaflexiviridae* genomes are encapsidated in filamentous virions and only genera *Botrexvirus* and *Sclerodarnavirus* infect fungi while the other five infect plants. (2) *Gammaflexiviridae* are very similar to *Alphaflexiviridae*, with genomes encapsidated in filamentous virions. The sole genus, *Mycoflexivirus*, exclusively infect fungi. Notably, *Alphaflexiviridae* and *Gammaflexiviridae* are the only mycoviral families that belong to an order, *Tymovirales*. (3) *Barnaviridae* (*ba*cilliform *RNA*) have genomes encapsidated in bacilliform virions and exclusively infect fungi. (4) *Hypoviridae* (*hypo* = “reduced” in Greek) have non-conventionally encapsidated genomes and are accommodated in host-derived lipid pleomorphic vesicles. Hypoviruses exclusively infect fungi and often cause significant hypovirulence. (5) *Narnaviridae* (*na*ked *RNA*) are the smallest, simplest known viruses with unencapsidated genomes. Genus *Mitovirus* are the most commonly found exclusively infect fungi and are localized in mitochondria. Genus *Narnavirus* infect fungi, oomycetes and protista and are localized in the cytoplasm. The positive-sense ssRNA mycoviruses that encode a reverse transcriptase enzyme and are incorporated into the hosts' genome are classified in two families, both forming round to ovoid virus like particles (VLPs): (1) In the *Metaviridae* genus *Metavirus* include species that infect fungi. (2) In *Pseudoviridae* genera *Hemivirus* and *Pseudovirus* include species that also infect fungi.

The family *Mymonaviridae* (*my*co + *mona*) and its sole genus *Sclerotimonavirus* (*scleroti* from *Scleroti**nia*, the host fungus, + *mona*) in the order *Mononegavirales* accommodate the only negative-sense ssRNA virus known to infect fungi, a linear molecule encapsidated in filamentous virions. Although the family exclusively infect fungi, the order *Mononegavirales* accommodate important human pathogenic viruses, including the family *Pneumoviridae* and the genera *Ebolavirus, Morbillivirus* (cause of measles) and *Lyssavirus* (cause of rabies).

Finally, the family *Genomoviridae*, genus *Gemycircularvirus* (*ge*mini-like + *my*co + *circular*) accommodate the only ssDNA virus known to infect fungi, a circular molecule encapsidated in isometric virions. Other members of the genus and the family were derived from environmental, plant-associated or animal-associated material^1^.

*Aspergillus* is a genus of filamentous fungi belonging to the class Eurotiomycetes, phylum Ascomycota and comprising >250 species. *Aspergillus* is one of the most well studied fungi with a worldwide distribution occupying numerous niches, while individual *Aspergillus* species are medically, ecologically and economically important (Rokas, 2013). Investigations on *Aspergillus* mycoviruses over the past 50 years have been extensive, leading to numerous ground-breaking discoveries and enhancing our understanding of viral diversity and the significance of virus infection/symbiosis in fungi.

In the light of the above, this review aims to present the current knowledge on *Aspergillus* mycoviruses, from their initial identification, population and transmission studies to their phenotypes, their molecular characterisation and the discovery of novel virus families.

## Incidence of mycoviruses in *Aspergilli*

The presence of mycoviruses in *Aspergillus* species was initially reported in 1970, when *Aspergillus foetidus* strain IMI 41871 and *A. niger* strain IMI 146891 were found to harbor spherical VLPs containing dsRNA (Banks et al., 1970). Notably these *A. foetidus* dsRNA elements were fully sequenced and characterized only recently (see “The *A. foetidus* mycovirus complex” section below). Since the 1980's screening large panels of *Aspergillus* isolates revealed the presence of dsRNA elements in various species (Tables 1, 2). Traditionally screening is performed by extracting total nucleic acids from the isolates of interest and visualizing extra, non-chromosomal bands by agarose electrophoresis. The dsRNA nature of these elements is demonstrated following enzymatic treatment with DNase I (degrades dsDNA), S1 nuclease (degrades ss nucleic acids), and RNase A in high salt conditions (degrades ssRNA) and low salt conditions (degrades ssRNA and dsRNA since the hydrogen bonds between the two RNA strands are more relaxed). The presence of VLPs associated with the dsRNA elements is shown by VLP purification and transmission electron microscopy (TEM).

**Table 1:** Incidence of dsRNA mycoviruses in *Aspergilli*.

	**Isolates screened**	**Isolates infected**	**Prevalence**	**References**
*Aspergillus* section Nigri	>300	35 (12%)	Indonesia/Tunis/India	Varga et al., 1994
	35	17 (50%)	Indonesia/Netherlands	van Diepeningen et al., 1997
	668	64 (10%)	worldwide	van Diepeningen et al., 2006
*Aspergillus* section Circumdati	27	4 (15%)	Hungary/Zair	Varga et al., 1998
*Aspergillus* section Flavi	92	10 (11%)	U.S.A	Elias and Cotty, 1996
*Aspergillus* section Clavati	8	2 (25%)	Hungary/U.S.A	Varga et al., 2003
*Aspergillus* section Fumigati	366	25 (7%)	U.K.	Bhatti et al., 2012
	86	16 (19%)	Netherlands	Refos et al., 2013

**Table 2:** *Aspergillus* strains harboring mycoviruses available from culture collections: American Type Culture Collection (ATCC), USA; CABI Bioscience, UK; Agricultural Research Service Culture Collection (ARSCC), USA; Centraalbureau voor Schimmelcultures (CBS), The Netherlands; Szeged Mycological Collection (SZMC), Hungary; National Institute of Hygienic Sciences (NHL), Japan; Brazilian Collection of Microorganisms from the Environment and Industry (CBM), Brazil; International Collection of Microorganisms from Plants (ICMP), New Zealand; Division of Food Research (FRR), Australia; Southern Regional Research Center (SRRC), USA.

**Section/species**		**ATCC**	**CABI**	**ARSCC**	**CBS-KNAW**	**OTHER**	**Mycovirus**	**References**
Nigri	*A. awamori*	ATCC 22342		NRRL 3112				Varga et al., 1994
	*A. flavus*	ATCC 26938		NRRL 5565 NRRL A12268				Wood et al., 1974; Gussack et al., 1977; Schmidt et al., 1986; Elias and Cotty, 1996
	*A. foetidus*	ATCC 10254	IMI 41871 IMI 15954	NRRL 337	CBS 126.48 CBS 618.78		*Aspergillus foetidus* complex (fully sequenced)	Banks et al., 1970; Varga et al., 1994; Kozlakidis et al., 2013a,b,c; Shah et al., 2015
	*A. foetidus*	ATCC 10254			CBS 126.48			Varga et al., 1994
	*A. foetidus*	ATCC 16874	IMI 104688		CBS 564.65		*Aspergillus foetidus* complex	
	*A. heteromorphus*	ATCC 12064	IMI 172288		CBS 117.55			Varga et al., 1994
	*A. niger*		IMI 146891				*Aspergillus niger* complex (antigenic cross-reactivity)	Banks et al., 1970
	*A. niger*		IMI 50555					Buck et al., 1973
	*A. niger*	ATCC 10577	IMI 27809	NRRL 2322 NRRL 2354				Varga et al., 1994
	*A. niger*				CBS 10366			Varga et al., 1994
	*A. niger*	ATCC 22343		NRRL 3122				Varga et al., 1994
	*A. niger*	ATCC 201254				SZMC 0585		Varga et al., 1994
	*A. niger*				CBS 223.43			van Diepeningen et al., 1998
	*A. tubingensis*	ATCC 201255				SZMC 0767		Varga et al., 1994
Circumdati	*A. ochraceus*	ATCC 28706					*Aspergillus ochraceus* complex (partially sequenced partitivirus)	Kim and Bozarth, 1985
	*A. ochraceus*	ATCC 1008	IMI 16247	NRRL 398 NRRL 1642	CBS 108.08 CBS 547.65			Varga et al., 1998
	*A. ochraceus*					ICMP 2043		Varga et al., 1998
	*A. petrakii*	ATCC 12337		NRRL 416	CBS 628.78			Varga et al., 2001
	*P. alliaceus*					FRR 4340		Varga et al., 1998
Flavi	*A. flavus*			NRRL 5918				Elias and Cotty, 1996
	*A. flavus*			NRRL 5940				Silva et al., 2001
	*A. leporis*		IMI 259100					Varga et al., 2001
	*A. nomius*			NRRL 5919				Elias and Cotty, 1996
	*A. parasiticus*					SRRC 75		Elias and Cotty, 1996
	*A. tamarii*			NRRL 20818				Elias and Cotty, 1996
Clavati	*A. clavatus*					SZMC 0918		Varga et al., 1998, 2003
	*A. clavatus*		IMI 358435					Varga et al., 2003
Fumigati	*A. fumigatus*					SZMC 4735		Varga et al., 2001
	*A. fumigatus*	ATCC MYA-4609			CBS 101355		AfuPmV-1 (fully sequenced)	Bhatti et al., 2012; Kanhayuwa et al., 2015
	*A. primulinus*					CBM-FA 0685		Varga et al., 2001
	*N. hiratsukae*					NHL 3008		Varga et al., 1998
	*N. hiratsukae*					NHL 3009		Varga et al., 1998
	*N. hiratsukae*		IMI 349860					Varga et al., 1998
	*N. quadricincta*	ATCC 16897		NRRL 2154				Varga et al., 1998

### *Aspergillus* section nigri

Black *Aspergilli*, or members of the *Aspergillus* section *Nigri*, are ubiquitous and include some of the most common *Aspergillus* species, such as *A. niger*. These fungi play an important role in agriculture and the food industry as plant infectious agents and food contaminants, together with medicine as a causative agent of aspergillosis. Black *Aspergilli* are also used in the fermentation industry to produce enzymes and chemicals (Abarca et al., 2004). Screening of >300, mostly Indonesian isolates revealed the presence of dsRNA elements, ranging in size from 0.5 to 5 kbp, in thirty five (ca. 12%) black *Aspergilli* isolates, including eleven *A. niger*, five *A. japonicus*, three *A. foetidus*, three *A. tubingensis*, two *A. heteromorphus*, one *A. carbonarius*, and one *A. awamori*. The electrophoretic profiles of several isolates were identical or similar to that of *A. foetidus* strain IMI 41871, but homology was not examined. Isometric VLPs ca. 30–35 nm and occasionally 23–25 nm in size were observed in the Indonesian isolates (Varga et al., 1994). A subsequent investigation showed that almost half (17/35) black *Aspergilli* isolates, 11 *A. niger*, 3 *A. tubingensis*, 1 *A. japonicas*, and 1 *A. carbonarius*, harbor dsRNA elements. Similarly, all but one of the virus-infected isolates originated from Indonesia (van Diepeningen et al., 1997). Further and more comprehensive screening revealed that 64/668 (ca. 10%) black *Aspergilli* isolates from all over the world were found to harbor up to eight dsRNA elements, ranging in size from 0.8 to 4.4 kbp encapsidated in isometric particles 25–40 nm in diameter (van Diepeningen et al., 2006).

The presence of mycoviruses has also been reported in several other *Aspergillus* species, including members of the sections Circumdati, Flavi, Clavati and Fumigati.

### *Aspergillus* section circumdati

These *Aspergilli* are often used for biochemical transformations; they produce proteolytic enzymes, metabolites, mycotoxins and promising anti-cancer compounds (Visagie et al., 2014). In *A. ochraceus* the presence of mycoviruses was initially reported by Kim and Bozarth (1985), who discovered isometric VLPs 30–32 nm in diameter containing 9 segments of dsRNA. Subsequently, 4/27 (15%) *A. ochraceus* isolates examined contained dsRNA elements ranging in size from 0.4 to 7 kbp (Varga et al., 1998). The presence of dsRNAs and VLPs 36–40 nm in diameter was also noted in *A. petrakii* (Varga et al., 2001).

### *Aspergillus* section flavi

Several species are used in food fermentation and biotechnological applications; importantly, *A. flavus* and other species in this section produce aflatoxins (Varga et al., 2011). *A. flavus* isolate NRRL 5565 contains isometric particles 27 and 30 nm in diameter (Wood et al., 1974; Gussack et al., 1977; Schmidt et al., 1986) and three dsRNA elements each ca. 3 kbp in size (Schmidt et al., 1986). Isolate NRRL 5940 also harbors three dsRNAs, approximately 3.7, 3.4, and 2.9 kbp in size and encapsidated in isometric particles 33 nm in diameter (Silva et al., 2001). Subsequently, 10/92 (11%) isolates of the *Aspergillus* section Flavi, five *A. flavus*, three *A. tamarii*, one *A. nomius* and one *A. parasiticus*, were found to contain 1–9 dsRNA elements 0.4–10 kbp in size (Elias and Cotty, 1996). The presence of dsRNAs and VLPs 36–40 nm in diameter was also noted in *Aspergillus leporis* (Varga et al., 2001).

### *Aspergillus* section clavati

These Aspergilli are significant food contaminants, since they produce numerous mycotoxins including patulin (Varga et al., 2007). In total, 2/8 (25%) *A. clavatus* strains were found to harbor isometric particles 35–40 nm in diameter (Varga et al., 2003). The monopartite 6 kbp dsRNA genome within the particles was considered an indication that these mycoviruses belong to the genus Totivirus, but this assumption has not been confirmed by sequence analysis.

### *Aspergillus* section fumigati

A. fumigatus is one of the most important human fungal pathogens, which causes aspergillosis in immunocompromised patients (Brakhage and Langfelder, 2002). In total, 25/366 (333 clinical and 33 environmental; ca. 7%) A. fumigatus isolates contained dsRNA elements (Bhatti et al., 2012). The electrophoretic pattern for seven of the isolates was characteristic for members of the family Partitiviridae (two dsRNAs, 1.5–2.0 kbp in size) and identical to that of *Aspergillus fumigatus* partitivirus-1 (AfuPV-1; Bhatti et al., 2011a). Similarly, five isolates contained four dsRNAs of a size and profile characteristic for members of the family Chrysoviridae and identical to that of *Aspergillus fumigatus* chrysovirus (AfuCV; Jamal et al., 2010). Finally, fourteen isolates contained four dsRNAs 1–2.5 kbp in size with a pattern identical to *Aspergillus fumigatus* tetramycovirus-1 (AfuTmV-1; Kanhayuwa et al., 2015) were found. Occasionally, some isolates appeared to be infected with more than one mycovirus (Bhatti et al., 2012). In another study, 16/86 (19%) clinical A. fumigatus isolates were found to harbor three to five dsRNA elements, ranging in size from 0.87 to 3 kbp (Refos et al., 2013).

### Sexual *Aspergilli*

Although mycoviruses are common in asexual Aspergilli, they have been reported only rarely in sexual Aspergilli. More specifically, 1/5 Petromyces alliaceus (the teleomorph of *A. alliaceus*) strains examined was shown to harbor two dsRNA elements 3.5 and 0.6 kbp in size where interestingly, the small element was localized in the mitochondrial fraction (Varga et al., 1998). Additionally, dsRNA elements and VLPs 36-40 nm in size were also observed in an A. primulinus isolate (Varga et al., 2001). In contrast, no dsRNA elements were discovered in a population study of 112 sexual *Aspergillus nidulans* isolates from Europe, Africa, America and Asia (Coenen et al., 1997). Finally, all three *Neosartorya hiratsukae* strains and 1/3 N. quadricincta strains exhibited similar but not identical patterns of dsRNA elements ranging in size from 1.3 to 3.4 kbp (Varga et al., 1998).

The presence and the quantity of dsRNA elements in *Aspergilli* have been reported to be dependent on the composition of the growth medium (Elias and Cotty, 1996). Since the dsRNA elements and the VLPs could not be detected in young mycelia (<48 h growth) and hyphal tips, it was suggested that at least some of these viruses multiply during the later phases of the fungal life cycle (Varga et al., 1994). In contrast, Silva et al. (2001) report that in *A. flavus* strain NRRL 5940 the number of viral particles increased during the exponential phase of growth and decreased during the later phases. No association between the presence of dsRNAs and the isolates' mitochondrial RFLP haplotype (van Diepeningen et al., 2006), specific conserved surface protein types or mating type MAT1-1 and MAT1-2 (Refos et al., 2013) have been observed.

## Horizontal and vertical mycovirus transmission

An extracellular phase in their replication cycle has not been reported for the majority of known mycoviruses, which spread vertically via production of sexual and asexual spores and horizontally via hyphal anastomosis and heterokaryosis. This lack of extracellular transmission route is attributed to the physical barrier of the fungal cell wall that generally does not allow for the direct uptake of viruses. However, some exceptions have been noted in recent years; for instance, Sclerotinia sclerotiorum hypovirulence-associated DNA virus 1, a gemycircularvirus, has both an extracellular phase being infectious as viral particles (Yu et al., 2013) and a transmission vector, the mycophagous insect *Lycoriella ingenua* (Liu et al., 2016).

In *Aspergilli*, the transfer of dsRNA elements to asexual conidia is very efficient (Coenen et al., 1997; van Diepeningen et al., 1997; Varga et al., 1998); however this is not the case for sexual ascospores. Coenen et al. (1997) in an attempt to explain the paucity of dsRNA elements in *Emericella nidulans*, the teleomorph of *A. nidulans*, established that the elements are not transmitted via ascospores unless ascospores were produced by self-fertilization. This observation was subsequently confirmed for *N. hiratsukae*, a species phylogenetically close to *Aspergillus* (Varga et al., 1998).

Of particular interest is how the viruses are transmitted from one fungal strain to another and the role of heterokaryon incompatibility between fungal isolates as a barrier to mycovirus spread. Varga et al. (1994) demonstrated that viruses can be transferred between isogenic or vegetatively compatible *A. niger* strains using polyethylene-glycol protoplast fusion. Occasionally not all dsRNA elements of the donor were transferred successfully to the recipient. Similar experiments were performed by van Diepeningen et al. (1997, 1998), confirming the restrictions posed by vegetative incompatibility and applying mechanical disturbance of mycelia to allow virus transfer between incompatible strains via the released cytoplasm. Interestingly, vegetative compatibility appeared to be irrelevant to mycovirus spread between *A. nidulans* strains (van Diepeningen et al., 1998); however, as described for the plant pathogenic ascomycete *Sclerotinia homoeocarpa*, different levels of (in)compatibility between fungal strains have been recognized, with distinct effects on mycovirus transmission (Deng et al., 2002). Additionally, mycovirus transmission has been correlated with the number of different vegetative incompatibility genes between the strains tested in another plant pathogenic ascomycete, *Cryphonectria parasitica* (Liu and Milgroom, 1996). Different viruses can co-exist in the same host (van Diepeningen et al., 1998), but also limiting competition among the mycoviruses was reported since extant infected isolates could not accommodate additional dsRNA elements (van Diepeningen et al., 1997).

Regarding intraspecies transmission, it has been successfully demonstrated between *A. niger* (donor) and *A. oryzae* and *A. ficuum* (recipients) by Liang and Chen (1987), *A. niger* (donor) and *A. tubingensis* (recipient) by Varga et al. (1994) and van Diepeningen et al. (1998), *A. niger* (donor) and *A. nidulans* (recipient) by Coenen et al. (1997) and van Diepeningen et al. (1998), *A. nidulans* (donor) and *A. niger* (recipient) by van Diepeningen et al. (1998), supporting the notion that these viruses are not restricted in one species. Interestingly, they do not appear to be restricted in hyphomycetes either: Lhoas (1972) reported using a virus suspension from *A. niger* strain IMI 146891 to infect the unicellular ascomycete *Saccharomyces cerevisiae*, a process successful solely during the mating of the yeast cells. The virus particles produced in the yeast cells post-infection were localized in vacuoles as aggregates or in the cytoplasm and appeared identical to the original ones in the hyphae of the filamentous fungus, confirming the ability of the virus to replicate in its new host (Border, 1972).

## Molecular characterisation of the *Aspergillus* mycoviruses

**Figure 2:**
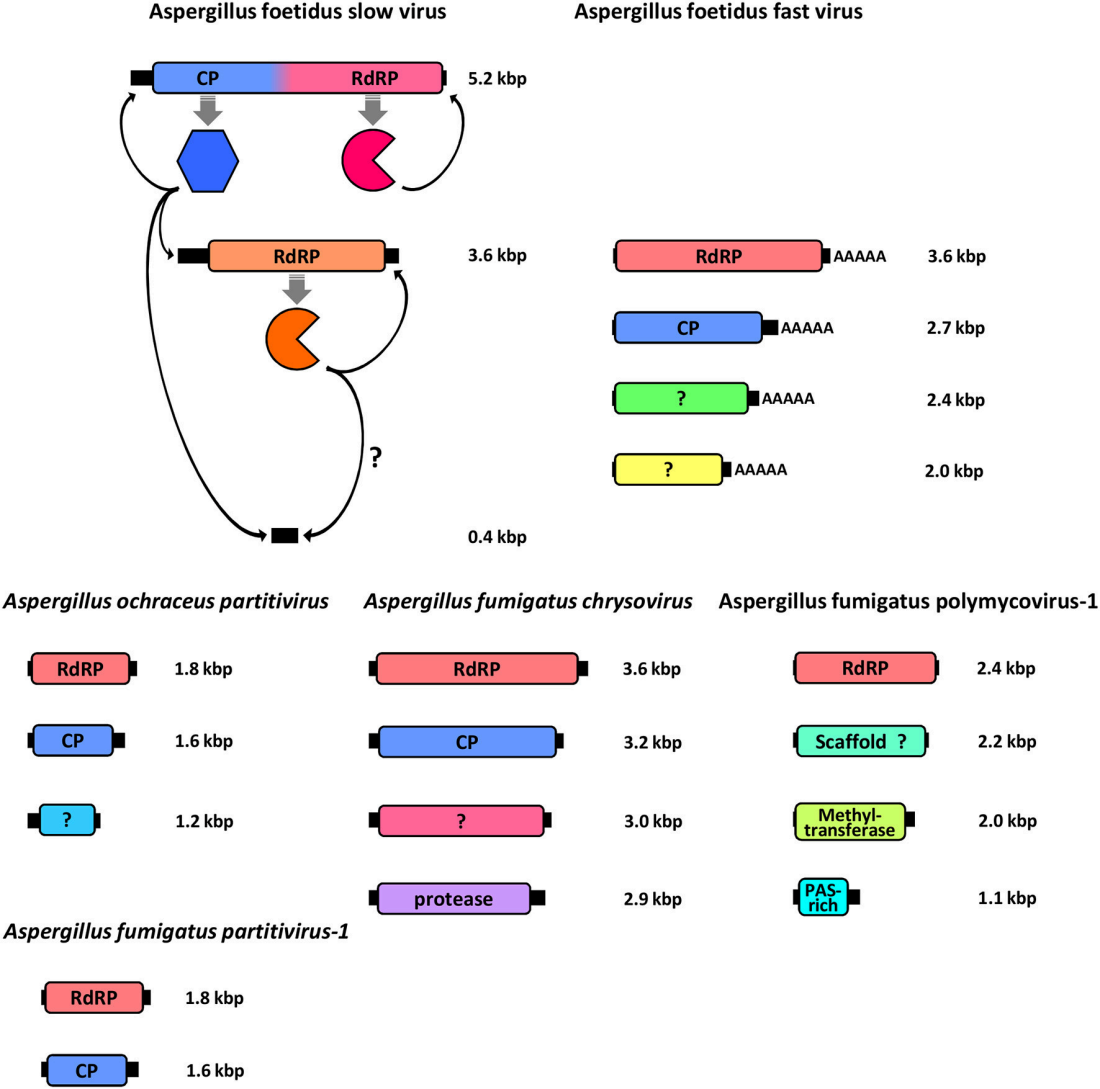
Schematic representation of the genomic organization of all fully sequenced *Aspergillus* mycoviruses, including the *A. foetidus* mycovirus complex, the *A. ochreacus* and *A. fumigatus* partitiviruses and the *A. fumigatus* chrysovirus and polymycovirus. For each genomic segment, the ORF(s) (colored boxes) are flanked by 5′- and 3′-UTRs (black boxes) and the function of the encoded protein(s) is indicated.

Despite the extensive population studies described in the previous section only a small number of mycoviruses found in *Aspergillus* have had their genomes fully sequenced and annotated. This information is crucial for assessing the diversity of the virome in *Aspergillus* species, since the electrophoretic profiles of the dsRNA elements on agarose gels cannot be reliably used to infer evolutionary relationships with other viruses. To date, known members of the *Aspergillus* virome belong to well-established families such as *Partitiviridae, Totiviridae* and *Chrysoviridae* and provisionally designated families such as Polymycoviridae and Alternaviridae that accommodate novel viruses (Figure 2, Table 3).

**Table 3:** Properties of known *Aspergillus* mycoviruses.

**Virus name**	**Abbreviation**	**Original host**	**Effects on host**	**Segment (bp)**	**Accession number**	**ORF size (nt; aa; kDa)**	**UTR length (bp)**	
							**5′-UTR**	**3′-UTR**
*Aspergillus ochraceus* partitivirus	AoPV	*A. ochraceus* FA0611	Unknown	dsRNA 1 (1,754)	EU118277	1,620; 539; 62	66	68
				dsRNA 2 (1,555)	EU118278	1,303; 433; 47	100	153
				dsRNA 3 (1,220)	EU118279	882; 293; 34	180	158
*Aspergillus fumigatus* partitivirus-1	AfuPV-1	*A. fumigatus* 88	Aconidial sectoring ↓ pigment ↓ radial growth ↓ biomass	dsRNA 1 (1,779) dsRNA 2 (1,623)	FN376847 FN398100	1,629; 542; 63 1,329; 442; 48	65 104	85190
*Aspergillus fumigatus* chrysovirus	AfuCV	*A. fumigatus* A-56	Aconidial sectoring ↑ pigment ↓ biomass	dsRNA 1 (3,560) dsRNA 2 (3,159) dsRNA 3 (3,006) dsRNA 4 (2,863)	FN178512 FN178513 FN178514 FN178515	3,345; 1,114; 129 2,862; 953; 107 2,681; 891; 99 2,544; 847; 95	128 167 169 154	87 130 156 165
*Aspergillus fumigatus* polymycovirus-1	AfuPmV-1	*A. fumigatus* Af293	Aconidial sectoring ↑ pigment ↑ virulence (*G. mellonella*)	dsRNA 1 (2,403) dsRNA 2 (2,233) dsRNA 3 (1,970) dsRNA 4 (1,131)	HG975302 HG975303 HG975304 HG975305	2,292; 763; 84 2,091; 696; 76 1,845; 614; 67 840; 279; 29	35 70 51 86	76 72 74 205
*Aspergillus foetidus* alternavirus	AfAltV	*A. foetidus* IMI 41871	Unknown	dsRNA 1 (3,571) dsRNA 2 (2,734) dsRNA 3 (2,418) dsRNA 4 (1,961)	HE588144 HE588145 HE588146 HE647818	3,375; 1,124; 127 2,406; 801; 87 2,181; 726; 79 1,764; 580; 65	51 48 50 50	145 280 187 147
*Aspergillus foetidus* victorivirus	AfVV	*A. foetidus* IMI 41871	Unknown	dsRNA 1 (5,194)	HE588147	4,745; 741/839; 78/92	373	76
*Aspergillus foetidus* exartavirus	AfExV	*A. foetidus* IMI 41871	Unknown	dsRNA 1 (3,634)	HE588148	2,889; 962; 110	509	236
*Aspergillus niger* victorivirus	AniVV	*A. niger* Ind 1.7.8	Unknown	dsRNA 1 (1,194/2,415)^*^	EU289894			
*Aspergillus niger* chrysovirus	AniCV	*A. niger* Ind 1.8.16	Aconidial sectoring ↓ spore production ↓ radial growth	dsRNA 1 (3,440)^*^	EU289896			
*Aspergillus niger* alternavirus	AniAltV	*A. niger* 341	Unknown	dsRNA 1 (3,576)	EU289897	3,375; 1,124; 127	51	145

* *Partial sequence*.

### The *Aspergillus foetidus* mycovirus complex

The dsRNA elements in *A. foetidus* strain IMI 41871 were among the first mycoviruses to be discovered (Banks et al., 1970) and constituted a model system in the 1970's for virus replication, transcription and biochemical studies (Ratti and Buck, 1975, 1978; Buck and Ratti, 1977), although the actual viruses were not sequenced and classified until recently.

*Aspergillus foetidus* strain IMI 41871 harbors seven dsRNA elements and two classes of VLPs. The VLPs were designated as fast (AfV-F) and slow (AfV-S) according to their electrophoretic mobility. Both AfV-F and AfV-S are isometric viruses, 33–37 nm in size (Ratti and Buck, 1972) and serologically unrelated to one another (Buck and Ratti, 1975). Each type is estimated to contain approximately 120 molecules of the respective coat protein (Buck and Ratti, 1975).

The AfV-F particles contain four dsRNAs, ca. 3.6, 2.8, 2.5, and 2.0 kbp in size, respectively, each one encapsidated in separate virions. These four dsRNAs constitute the genome of a novel unclassified virus, provisionally designated as *Aspergillus foetidus* alternavirus (AfAV; Kozlakidis et al., 2013c). Each dsRNA has a single open reading frame (ORF), conserved 5′- and 3′-terminal sequences that may be involved in replication and a 3′ poly (A) tail ranging in length from 17 to 62 nt. The largest dsRNA encodes the viral RNA dependent RNA polymerase (RdRP), 127 kDa in size, and classified in one of the viral RdRP families (RdRP_4, pfam02123). The other three dsRNAs encode polypeptides of unknown function 87.5, 79, and 65 kDa in size, respectively (Kozlakidis et al., 2013c). The 87.5 kDa protein is probably the virus coat protein (CP) since its size corresponds to that of the major virion protein (Buck and Ratti, 1975).

The AfV-S particles contain three dsRNAs, ca. 5.2, 3.6, and 0.4 kbp in size, respectively. The largest dsRNA is provisionally designated as *Aspergillus foetidus* victorivirus-1 (AfVV-1) and its genomic organization is typical of members of the genus *Victorivirus*, family *Totiviridae*, with two overlapping ORFs expressed via a coupled termination/reinitiation mechanism. The upstream ORF encodes the CP, 78 kDa in size, while the downstream ORF encodes the viral RdRP, 92 kDa in size (Kozlakidis et al., 2013a). The second dsRNA element is provisionally designated *Aspergillus foetidus* Exartavirus (AfExV; *exarta-* = “dependent” (for encapsidation) in Greek), contains an ORF encoding an RdRP, 100 kDa in size, and an unusually long 5′-UTR (Kozlakidis et al., 2013b). The smallest dsRNA is a non-coding satellite RNA with a 5′-terminal sequence identical to that of AfExV (Shah et al., 2015) suggesting that both molecules are replicated by AfExV RdRP, while all three dsRNAs are encapsidated by the AfVV-1 CP.

The *Aspergillus foetidus* viruses were the first to be reported in *Aspergillus* sp. and appear to be rather common as identical or similar electrophoretic patterns have been reported for other isolates (Liang and Chen, 1987; Varga et al., 1994); however, without further molecular or biochemical evidence no definitive conclusions can be drawn. A similar virus complex has been described in *A. niger* isolates IMI 146891 (Banks et al., 1970) and IMI 50555 (Buck et al., 1973) and antigenic cross-reactivity between the *A. foetidus* and *A. niger* viruses was also demonstrated (Buck et al., 1973). Interestingly, in *A. niger* IMI 50555 the second largest dsRNA that would correspond to the exartavirus, is encapsidated by both AnV-F and AnV-S particles, a phenomenon called genomic masking (Buck et al., 1973).

Individual viruses comprising the *A. foetidus* complex have been discovered in other fungal species as well. For instance the family *Totiviridae* is well established and contains the genus *Victorivirus* comprising fourteen official members plus the partially sequenced dsRNA *Aspergillus niger* victorivirus-1 (AniVV-1) *ca*. 6 kbp in size isolated from *A. niger* strain Ind 1.7.8 (Hammond et al., 2008). In this investigation two fragments 1.2 and 2.4 kbp is size are significantly similar in sequence to the CP and RdRP genes of respectively Magnaporthe oryzae virus 3 and Penicillium aurantiogriseum totivirus 1, both established victoriviruses.

**Figure 3:**
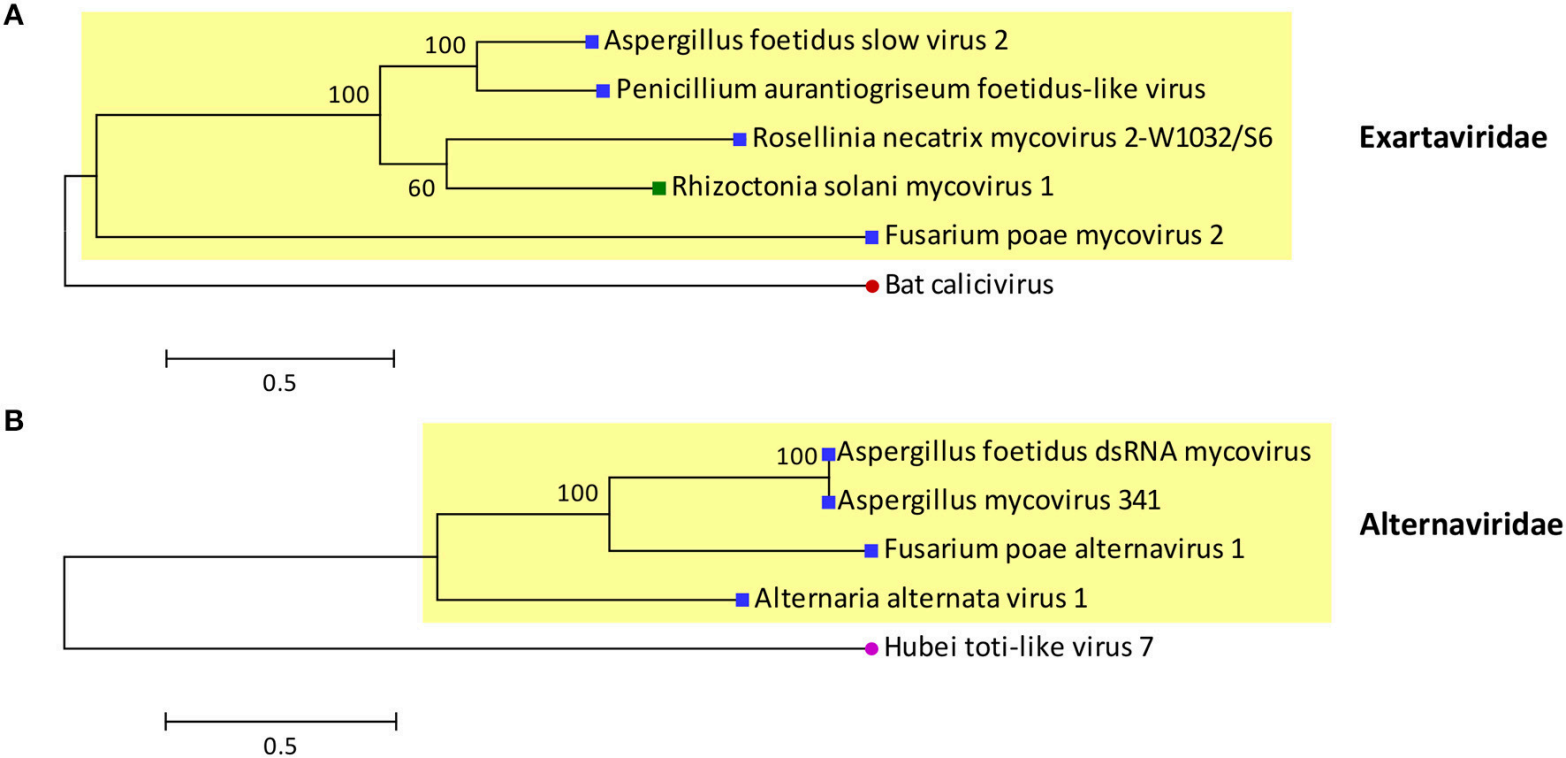
Phylogenetic analysis of the proposed families **(A)** Exartaviridae and **(B)** Alternaviridae. Putative exartaviruses (RdRP_1, PF00680; Finn et al., 2014) include *Aspergillus foetidus* slow virus 2 (CCD33025), Penicillium aurantiogriseum *foetidus*-like virus (YP_009182156), Rosellinia necatrix mycovirus 2-W1032/S6 (BAT50982), Rhizoctonia solani mycovirus 1 (ANR02697), and Fusarium poae mycovirus 2 (YP_009272910). A bat calicivirus (AIF74264), the most closely related virus (PSI-BLAST; Altschul et al., 1997), was used as an outgroup. Putative alternaviruses (RdRP_4, PF02123; Finn et al., 2014) include *Aspergillus foetidus* dsRNA mycovirus (YP_007353985), *Aspergillus* mycovirus 341 (ABX79997), Fusarium poae alternavirus 1 (YP_009272952) and Alternaria alternata alternavirus-1 (YP_001976142). Hubei toti-like virus 7 (APG76025), the most closely related virus (PSI-BLAST; Altschul et al., 1997), was used as an outgroup. The RdRP sequences of the viruses were aligned with MUSCLE as implemented by MEGA 6 (Tamura et al., 2013) the alignment was improved manually and all positions with less than 30% site coverage were eliminated. Maximum likelihood phylogenetic trees were constructed with MEGA 6 using the WAG+G+I+F and LG+G substitution models for exartaviruses and alternaviruses respectively. At the end of the branches blue and green squares indicate that the virus infects ascomycetes and basidiomycetes respectively; red and purple circles indicate that the virus infects mammals and invertebrates respectively.

The proposed family Exartaviridae would include AfEx, Penicillium aurantiogriseum *foetidus*-like virus (Nerva et al., 2016), Rosellinia necatrix mycovirus 2-W1032/S6 (Zhang et al., 2016) and Fusarium poae mycovirus 2 (Osaki et al., 2016), isolated from ascomycetes, and Rhizoctonia solani mycovirus 1 (Bartholomäus et al., 2016), isolated from a basidiomycete (Figure 3). Interestingly, the exartaviruses have been argued to be positive-sense ssRNA viruses with a dsRNA replicative form (Zhang et al., 2016). The proposed family Alternaviridae includes Alternaria alternata alternavirus-1 (Aoki et al., 2009) as its prototype member, AfAV and Fusarium poae alternavirus 1 (Osaki et al., 2016), both isolated from ascomycetes. Additionally, another alternavirus from *Aspergillus* has been partially sequenced (Hammond et al., 2008); *Aspergillus niger* alternavirus (AniAltV), originally isolated from *A. niger* strain 341, has four dsRNA segments, ranging in size from 1.5 to 3.6 kbp, as its genome where the largest was sequenced and found encode an RdRP with 99% similarity to AfAltV RdRP.

### Partitiviruses in *Aspergillus*

**Figure 4:**
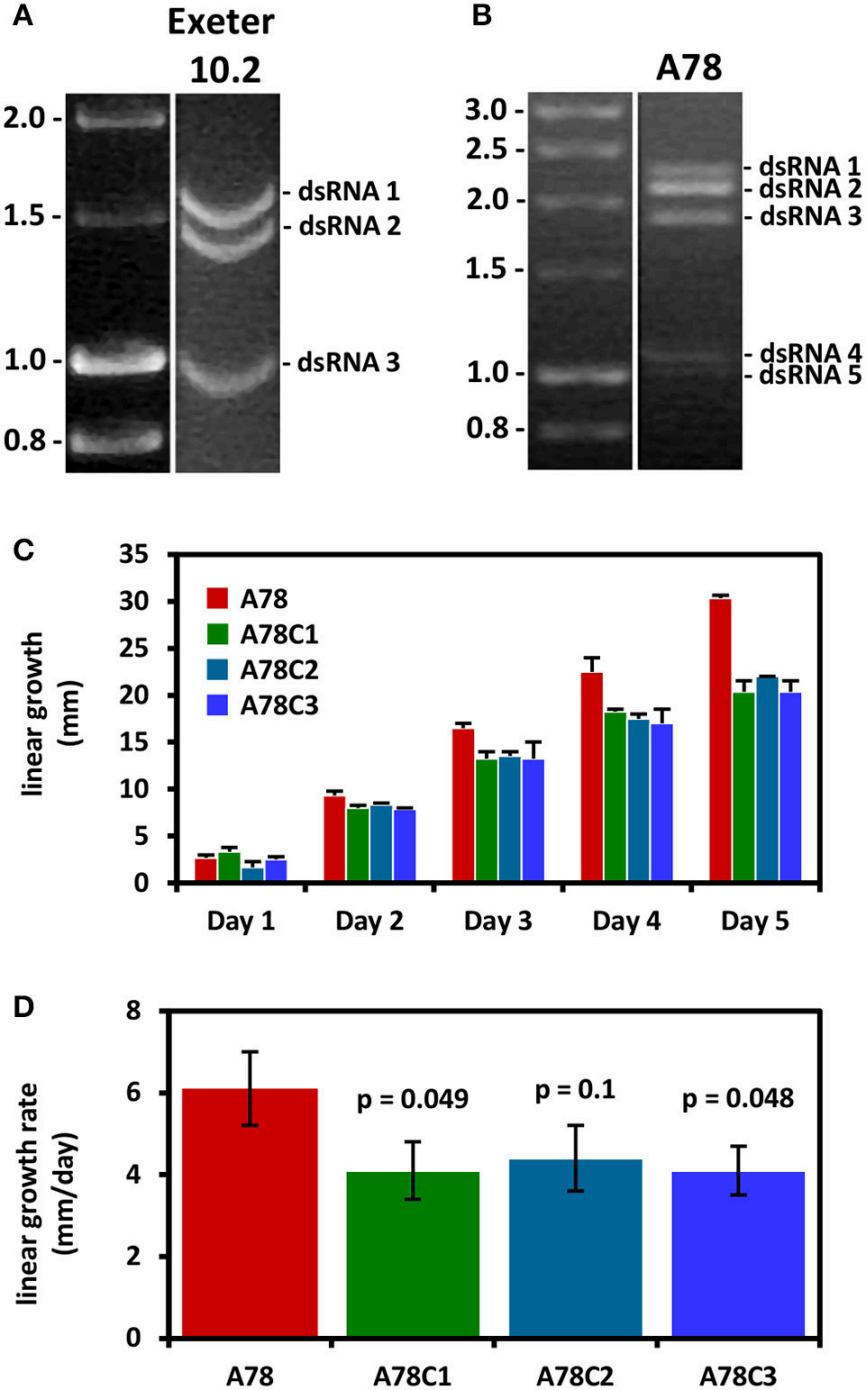
**(A)** Agarose gel electrophoresis of dsRNA molecules from *A. fumigatus* strain Exeter 10.2 harboring a partitivirus. The dsRNAs 1 and 2 encode the RdRP and CP, respectively, while dsRNA 3 is an additional genomic component (adapted from Bhatti, 2011). **(B)** Agarose gel electrophoresis of dsRNA molecules from *A. fumigatus* strain A78, harboring a polymycovirus. A fifth dsRNA molecule is visible under the four dsRNAs constituting the viral genome (adapted from Readman, 2011). **(C,D)** Three virus-free isogenic colonies derived by curing the virus-infected *A. fumigatus* strain A78 exhibit reduced radial growth and radial growth rate on solid minimal medium. The *p*-values indicate the statistical significance of the difference in growth rate between the virus-infected and the three cured virus-free strains (Student's *t*-test; adapted from Readman, 2011).

Two members of the genus *Gammapartitivirus*, family *Partitiviridae* have been isolated from *Aspergillus, Aspergillus ochraceus virus* (AoV) from *A. ochraceus* strain FA0611 (Liu et al., 2008) and *Aspergillus fumigatus* partitivirus-1 (AfuPV-1) from *A. fumigatus* isolate 88; (Bhatti et al., 2011a). A strain of the former has been also found in *A. ochraceus* ATCC 28706 (Kim et al., 2006). Both viruses are typical members of their genus, with two dsRNA segments ca. 1.8 and 1.6 kbp in length as their genome, each containing one ORF. The large dsRNA encodes the viral RdRP, 62 and 63 kDa for AoV and AfuPV-1, respectively, while the small dsRNA encodes the CP, 47 and 48 kDa for AoV and AfuPV-1, respectively. For both viruses the 5′- and 3′-termini of untranslated regions (UTRs) flanking the ORF are highly conserved (Liu et al., 2008; Bhatti et al., 2011a). Interestingly, AoV has a third component, a dsRNA ca. 1.2 kbp in length that encodes a 34 kDa protein of unknown function. The UTRs of this dsRNA are very similar to those of the other two AoV components, suggesting that it is replicated by the RdRP (Liu et al., 2008). An additional dsRNA has also been reported for an AfuPV-1-like virus from the environmental *A. fumigatus* isolate Exeter 10.2, although it has not been sequenced (Bhatti, 2011; Figure 4A). Other gammapartitiviruses known to have a homologous third dsRNA are Gremmeniella abietina RNA virus MS1 (Botella et al., 2015), Gremmeniella abietina RNA virus MS2 (Tuomivirta and Hantula, 2005), and Ustilaginoidea virens partitivirus (Zhang et al., 2013), all of which infect ascomycetes.

### Chrysoviruses in *Aspergillus*

A member of the family *Chrysoviridae* has been isolated from *A. fumigatus* isolate A-56 by Jamal et al. (2010) and designated as *Aspergillus fumigatus chrysovirus* (AfuCV). The AfuCV genome consists of four dsRNAs, ca. 3.6, 3.2, 3.0, and 2.8 kbp in size, respectively, each one containing an ORF flanked by conserved 5′- and 3′-UTRs. The two largest dsRNAs encode the viral RdRP and CP, 128 and 107 kDa in size, respectively. AfuCV dsRNA3 and dsRNA4 encode two proteins 99 and 95 kDa homologous to those encoded by dsRNA4 and dsRNA3 of typical chrysoviruses, a phenomenon observed previously for *Penicillium chrysogenum virus* and Verticillium dahliae chrysovirus 1. The former protein contains a “phytoreo S7 domain,” shares significant sequence similarity with the N-terminus of the RdRP and is considered to interact with it and play a role in viral RNA binding and packaging (Ghabrial, 2008), while the latter is a putative protease. Additionally, another chrysovirus has been found in *A. niger* strain Ind 1.8.16. *Aspergillus niger* chrysovirus-1 (AniCV-1) and consists of four dsRNAs, ranging in size from 2 to 3.7 kbp, and one 3.4 kbp fragment derived from the largest dsRNA has been sequenced and found to encode an RdRP (Hammond et al., 2008).

### A polymycovirus in *Aspergillus fumigatus*

*Aspergillus fumigatus* polymycovirus-1 (AfuPmV-1) is the prototype member of the proposed family Polymycoviridae (Kotta-Loizou and Coutts, 2017), originally named Tetramycoviridae (Kanhayuwa et al., 2015), and was derived from *A. fumigatus* strain Af293. The AfuPmV-1 genome consists of four dsRNAs, similarly to the proposed family Alternaviridae and the established family *Chrysoviridae* described above; however they are smaller in size, ca. 2.4, 2.2, 2.0, and 1.1 kbp. The largest dsRNA encodes the viral RdRP, which is unique in that although related to the positive-stranded RNA picorna-superfamily has its catalytic GDD motif replaced with GDNQ, characteristic of negative-stranded RNA viruses. The second largest dsRNA encodes a protein of unknown function with a zinc finger-like motif and a putative ER retaining signal peptide. The third largest RNA encodes a methyl-transferase responsible for capping the positive RNA strands; notably this is the first report of a capping enzyme and the RdRP being on distinct dsRNA elements. Finally, the smallest dsRNA encodes a proline-alanine-serine (PAS) rich protein that is hypothesized to coat the viral genome, which is non-conventionally encapsidated. The virus was also shown to be infectious to *A. fumigatus* protoplasts as dsRNA (Kanhayuwa et al., 2015). Subsequently, members of the proposed family Polymycoviridae, such as those isolated from the insect pathogenic fungus *Beauveria bassiana* (Kotta-Loizou and Coutts, 2017) or the plant pathogenic fungus *Botryosphaeria dothidea* (Zhai et al., 2016), were discovered to have up to three additional small dsRNA elements encoding non-homologous proteins of unknown function. This is not the case for AfuPmV-1 but an uncharacterized virus with an identical electrophoretic profile isolated from *A. fumigatus* isolate A78 may harbor a fifth dsRNA segment (Readman, 2011; Figure 4B). Interestingly, a recently published related mycovirus from the plant pathogenic fungus *Colletotrichum camelliae* has four additional dsRNA segments, is also infectious as dsRNA and forms filamentous virions (Jia et al., 2017).

## Effects of mycoviruses on the fungal host

In order to reliably assess the effect of mycoviruses on their hosts it is necessary to produce virus-free and virus-infected isogenic lines, either by curing the virus-infected strain or by introducing the virus into a virus-free strain. Cycloheximide, a protein synthesis inhibitor, was successfully used to eradicate dsRNA mycoviruses from yeast (Fink and Styles, 1972) and subsequently from other fungi including *Aspergilli* (Elias and Cotty, 1996; Bhatti et al., 2011b). Cycloheximide-based infection curing is not always successful (Varga et al., 1998; van Diepeningen et al., 2006); Elias and Cotty (1996) reported a success rate of 40%, while Bhatti et al. (2011b) managed to eradicate AfuCV but not AfuPV suggesting that both individual fungal strains and viruses might have different sensitivities to cycloheximide. Similarly, 5′-fluorouracil has also been used in this context (Schmidt et al., 1986). Other methods that have been used for curing virus-infected fungi with various rates of success include single conidial isolation (Elias and Cotty, 1996; van Diepeningen et al., 2006), hyphal tipping (van Diepeningen et al., 2006) and selection for nitrogen-metabolism and other mutants (Varga et al., 1994; Elias and Cotty, 1996). Alternatively, isogenic lines can be produced by introducing the virus of interest into a virus-free strain, either via protoplast transfection or protoplast fusion. In examples where cycloheximide or other stress-inducing or mutagenic chemicals were originally used to eradicate the virus it is recommended the virus is reintroduced into the cured strain by protoplast transfection in order to ensure that any phenotypes observed are exclusively due to the presence of the virus.

The phenotypes commonly assessed by comparing virus-free and the virus-infected strains are pigmentation, growth, virulence and toxin production. Growth can be examined both in solid (radial growth) and in liquid (biomass production) medium, while growth media that differ in their nutritional values often serve to illustrate more clearly the effect of the virus on its host. To date, virulence in *Aspergilli* is determined using two model systems: the murine model, where the fungal burden in the lungs of immunosuppressed mice is measured by quantitative polymerase chain reaction, and larvae of the greater wax moth *Galleria mellonella*, where both survival rates and fungal burden are estimated (Özkan and Coutts, 2015).

In *A. flavus* strain NRRL 5565, the production of carcinogenic aflatoxins was shown to be repressed by the presence of a mycovirus (Schmidt et al., 1986). Similarly, in strain NRRL 5940, aflatoxin production occurred in parallel with decreased presence of viral particles (Silva et al., 2001). However Elias and Cotty (1996) reported that there was no correlation between the presence of dsRNA and the production of aflatoxin in other *A. flavus* isolates. In *A. clavatus* strains, the presence of mycoviruses was not associated with the production of the mutagenic patulin either (Varga et al., 2003). Since these mycotoxins, which are found on agricultural crops and may be consumed either directly or indirectly via meat and dairy products, represent a significant medical and economic issue especially in developing countries (Kumar et al., 2017), the idea of controlling their production using mycoviruses is very appealing. Unfortunately no further studies were performed to characterize the dsRNAs from the non-aflatoxin producing strain at the molecular level.

Virus-infected *A. niger* strain Ind 1.8.16 was observed to form aconidial sectors that demonstrated a reduced growth rate and increased dsRNA levels. A direct comparison of virus-infected and virus-free isogenic lines confirmed that the presence of dsRNA was associated with a statistically significant decrease in radial growth rate, spore production and competitive spore production that was more evident on minimal medium when compared to water agar, very minimal medium and complete medium (van Diepeningen et al., 2006).

The AfuCV-infected *A. fumigatus* isolate A-56 produces darker green pigmentation than the AfuCV-free isogenic strain and forms aconidial sectors. Additionally, its biomass in both minimal and *Aspergillus* complete liquid medium is significantly smaller (Bhatti et al., 2011b); however no statistically significant differences in virulence were observed between the AfuCV-free and the AfuCV-infected strain using the murine infection model (Bhatti et al., 2011b) and *G. mellonella* (Özkan and Coutts, 2015). Similarly, the AfuPV-1-transfected *A. fumigatus* isolate 237y demonstrated aconidial sectoring and lighter pigmentation when compared to the AfuPV-1-free isogenic strain, together with a significantly slower radial growth and biomass production in both minimal and *Aspergillus* complete medium (Bhatti et al., 2011b). Again no statistically significant differences in virulence were observed in the murine (Bhatti et al., 2011b) or *G. mellonella* (Özkan and Coutts, 2015) infection models. Comparison of the AfuPmV-1-infected *A. fumigatus* isolate 293 with a AfuPmV-1-free isogenic strain revealed no statistically significant differences in radial growth rate or biomass production; however a statistically significant decrease in the survival rates of *G. mellonella* larvae infected with the AfuPmV-1-infected strain was observed. This was not accompanied with a concomitant increase in the fungal burden, suggesting that the hypervirulent effect is not due to fungal growth (Kanhayuwa et al., 2015). *A. fumigatus* isolate A78 harbors an uncharacterised virus with an electrophoretic profile identical to that of AfuPmV-1. Both virus-infected isolates show a minor increase in the intensity of pigmentation and form sectors (Kanhayuwa et al., 2015; Özkan and Coutts, 2015); however, in the case of isolate A78, the virus-infected strain demonstrated a statistically significant increase in radial growth rate that was more prominent in minimal than in *Aspergillus* complete medium. This was independently confirmed using three distinct cured isogenic virus-free colonies (Readman, 2011; Figure 4C). Additionally, a statistically significant decrease in the survival rates of *G. mellonella* larvae exposed to the virus-infected A78 isolate was noted, which was probably due to the faster growth of the strain as indicated by the fungal burden assay (Özkan and Coutts, 2015). Given the medical significance of *A. fumigatus* infections and the hypo- or hyper-virulent effects of the viruses on their host, it would be worthwhile to investigate further the presence of distinct mycoviruses in clinical samples in association with clinicopathological parameters and patient survival. Additionally, any potential links between mycoviruses and the worldwide problem of azole resistance (Perlin et al., 2017) should be elucidated. Aspergillosis is observed in patients with hematological malignancies or those who have undergone organ transplantation and, in the case of azole resistant strains, is associated with mortality rates up to 100% (Meis et al., 2016).

The molecular mechanisms underpinning these phenotypes in *Aspergillus* are still largely unexplored. However almost a decade ago, Hammond et al. (2008) indicated that at least some mycoviruses (as exemplified by AniCV) may suppress RNA silencing in *A. nidulans* and some others, such as the non-conventionally encapsidated AniAltV, may be silenced themselves as part of the anti-viral defense of their host strains. RNA silencing as an anti-mycoviral defense was initially studied in *C. parasitica*, where deletion of important genes encoding dicer-like or Argonaut-like proteins could lead to severe growth defects in the virus-infected but not the virus-free strains (Segers et al., 2007; Sun et al., 2009). Additionally, the papain-like protease encoded by Cryphonectria hypovirus 1 was identified as a RNA silencing suppressor both in fungi and plants (Segers et al., 2006). More recently, Özkan et al. (2017) demonstrated using high throughput small (s) RNA sequencing that non-conventionally encapsidated viruses, such as AfuPmV-1, and to a lesser extent encapsidated viruses, such as AfuPV-1 and AfuCV, are silenced in *A. fumigatus*. More specifically, 50% of total sRNAs where derived from AfuPmV-1, while the percentage was approximately half, 25 and 22% for AfuPV-1 and AfuCV, respectively. Additionally, sRNAs differentially expressed in virus-free and virus-infected samples were identified, suggesting a putative pathway for mediating observed mycovirus effects on fungal hosts (Özkan et al., 2017).

## Conclusion

Mycoviruses in *Aspergilli* are more extensively studied than in any other fungal genus. The major established viral families, including *Partitiviridae, Chrysoviridae*, and *Totiviridae*, are well represented in *Aspergillus* species, while novel families such as Polymycoviridae, Alternaviridae and Exartaviridae have been proposed based on discoveries made in *Aspergilli*. Population studies and basic mycovirological research have provided valuable insights into the distribution and transmission of mycoviruses in medically, ecologically and economically important species. Finally, distinct phenotypes have been associated with the presence of mycoviruses although their molecular mechanisms are still unknown.

Future studies should focus on elucidating the molecular mechanisms of host-mycovirus interactions, especially the effects of the mycovirus infection on the fungal host. Off-target RNA silencing is expected to play a role given the dsRNA nature of these viruses that act as both triggers and targets of the antiviral defense system. Additionally, host-virus protein-protein interactions via Eukaryotic Linear Motifs leading to disruption of host pathways may also be implicated in accordance with other host-pathogen systems. The mechanisms controlling mycovirus-induced phenotypic alterations, from reduced aflatoxin expression to hyper- or hypo-virulence are still unclear but such knowledge would facilitate genetic engineering of known mycoviruses in order to induce desired phenomena. The discovery of non-conventionally encapsidated viruses which are infectious as dsRNA, such as polymycoviruses, will facilitate the process by providing us with tractable systems to study and engineer at will.

## Author contributions

All authors listed have made a substantial, direct and intellectual contribution to the work, and approved it for publication.

### Conflict of interest statement

The authors declare that the research was conducted in the absence of any commercial or financial relationships that could be construed as a potential conflict of interest.

## Footnotes

^1^All the information on virus families was derived from Virus Taxonomy: The Classification and Nomenclature of Viruses – The Online (10th) Report of the ICTV; https://talk.ictvonline.org/ictv-reports/ictv_online_report/.
